# Spontaneously Resolving Pneumoperitoneum and Pneumomediastinum: A Report of Two Cases

**DOI:** 10.7759/cureus.61930

**Published:** 2024-06-08

**Authors:** Wardah Mahmood, Mansoor Zafar, Stefano Berliti, Ariful Islam Islam, Viktoriya Clarke, Kadir Hacikurt

**Affiliations:** 1 Medicine, Conquest Hospital - East Sussex Healthcare NHS Trust, St. Leonards-on-sea, GBR; 2 Gastroenterology, Hammersmith Hospital - Imperial College Healthcare NHS Trust, London, GBR; 3 Acute Medicine, Conquest Hospital - East Sussex Healthcare NHS Trust, St. Leonards-on-sea, GBR; 4 Radiology, Conquest Hospital - East Sussex Healthcare NHS Trust, St. Leonards-on-sea, GBR

**Keywords:** pneumomediastinum, pneumoperitoneum, iatrogenic effect, computed tomogram, spontaneous subcutaneous emphysema

## Abstract

We present here an interesting case report of two patients with spontaneous pneumomediastinum and iatrogenic pneumoperitoneum. The patients were assessed and queried following a chest X-ray abnormality and query based on the history of recent urological procedures on a background of awaiting gastro-oesophageal surgery at a tertiary centre respectively. Although these patients were successfully managed with the best supportive approach and periodic imaging review, it remains important to be aware that fatalities have been reported in the literature. We hope this case report will help those involved in the care of the patient to be aware of these conditions as differentials when history points towards episodes of coughing or recent surgical input.

## Introduction

Pneumoperitoneum is defined as the presence of air in the peritoneum within the abdominal cavity. Although it can be detected with abdominal X-ray, if pneumoperitoneum is in small amounts, it may be missed in which case computed tomogram (CT) remains the investigation of choice to confirm [1]. The commonly reported causes in adults include peritoneal dialysis, operative and immediate post-operative status, vaginal aspiration, and bowel perforation following colonoscopy, endoscopy, and endoscopic retrograde cholangiogram. Occasionally pneumomediastinum and pneumothorax may lead to pneumoperitoneum [1,2]. The pneumoperitoneum has been classified as surgical pneumoperitoneum and reported to represent 85-90% of all pneumoperitoneum cases, and the non-surgical occurrence has been reported to account for 5-15% of all occurrences [3].

Pneumomediastinum also described as mediastinal emphysema is defined as the presence of air in the mediastinum [4-6]. Interestingly, when this is associated with subcutaneous emphysema it is known as Hamman's Syndrome and was first described in 1939 by Hamman in a post-partum patient [7], although the phenomenon has been reported to have been first described by Laenek in 1827 [4-6]. 

We present an interesting case report of two patients who presented to the emergency department and were referred to the medical on-call team within two months. Both patients had completely different comorbidities and one of them was found to have pneumoperitoneum and the other patient with pneumomediastinum two months later. This case report highlights the importance of a holistic approach with clinical assessment and the judicial use of imaging modalities to diagnose and aid in a prompt management plan. 

## Case presentation

Case 1: pneumoperitoneum

A 67-year-old non-smoker male with a body mass index (BMI) of 29 presented to the emergency department (ED) with concerns of progressively worsening vague generalised abdominal pain. He had a background history of type 2 diabetes mellitus (DM), sleep apnoea, atrial fibrillation, previous sphincterotomy for the common bile duct stones and a recent oesophageal adenocarcinoma diagnosis along the gastro-oesophageal junction undergoing chemotherapy and waiting for radical surgery for excision at a tertiary centre. With difficulties in swallowing, he had percutaneous endoscopic gastrostomy (PEG) tube insertion to meet his ongoing nutrition needs. Additionally, four weeks before this visit to the hospital's ED, he underwent a four-robotic arm technique laparoscopy for the excision of a 6cm right renal tumour extending into the renal vein. This was achieved following establishing a pneumoperitoneum with a carbon dioxide (CO_2_) insufflation pressure of 15mmHg while the other ports were placed under direct vision. However, the specimen was noticed to be too big and hence extracted manually via transverse incision. Post-operatively, the patient stayed briefly in a high-definition unit setting till recovery post-urological surgery.

**Figure 1:**
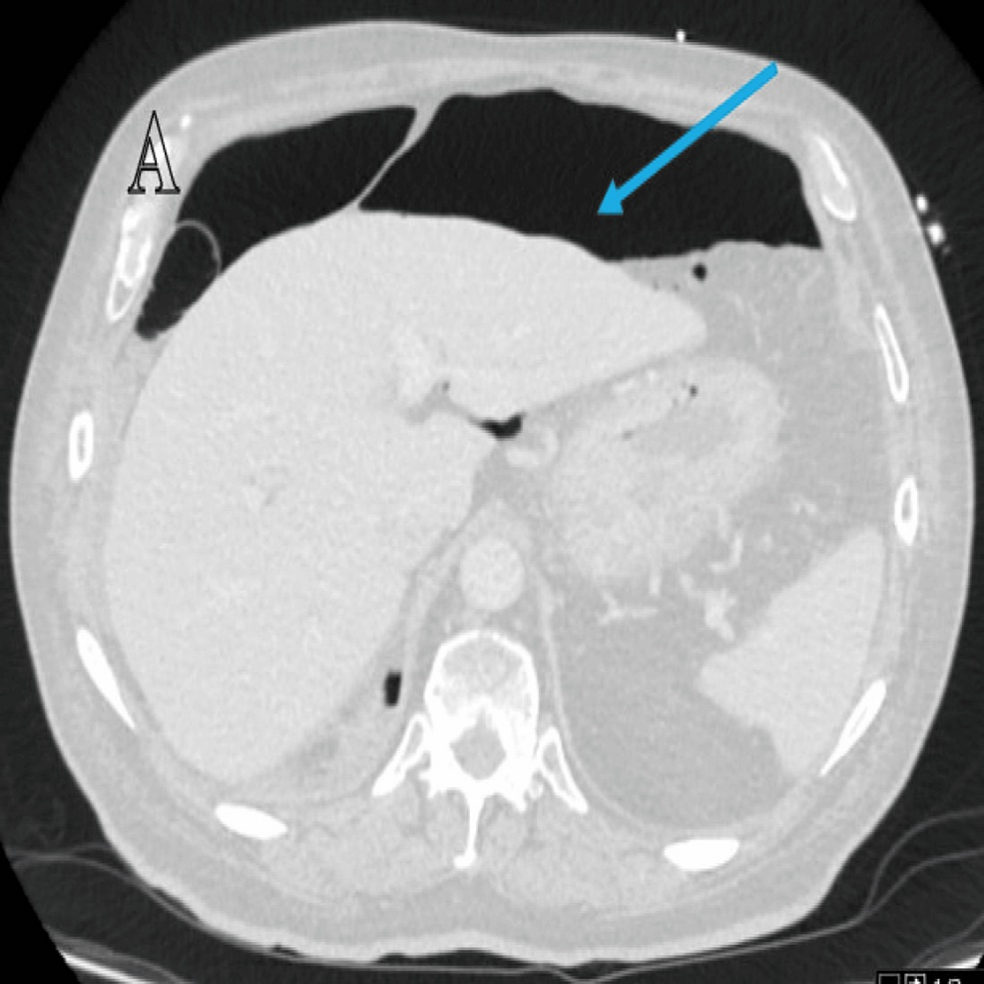
Case 1: Axial computed tomogram (CT) image showing extensive pneumoperitoneum (blue arrow).

The patient was referred to the medical on-call team and during the examination, he was noticed to have vague generalised tenderness with no guarding or rebound tenderness. The PEG site appeared secured with no oozing or leaks and his vital signs (observations) were stable. He underwent a computed tomogram (CT) scan of the abdomen and pelvis to investigate the cause which showed a massive pneumoperitoneum (Figure 1).

**Figure 2:**
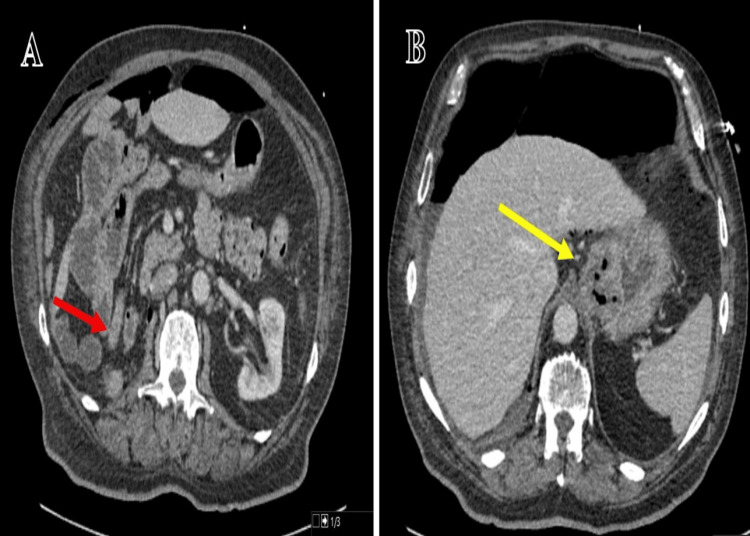
Case 1: Axial computed tomogram (CT) image showing right nephrectomy - (A) (red arrow) and thickening of the gastroesophageal junction - (B) (yellow arrow).

His CT scan also showed thickening along the lower gastroesophageal junction and the site of the previous nephrectomy (Figure 2).

**Figure 3:**
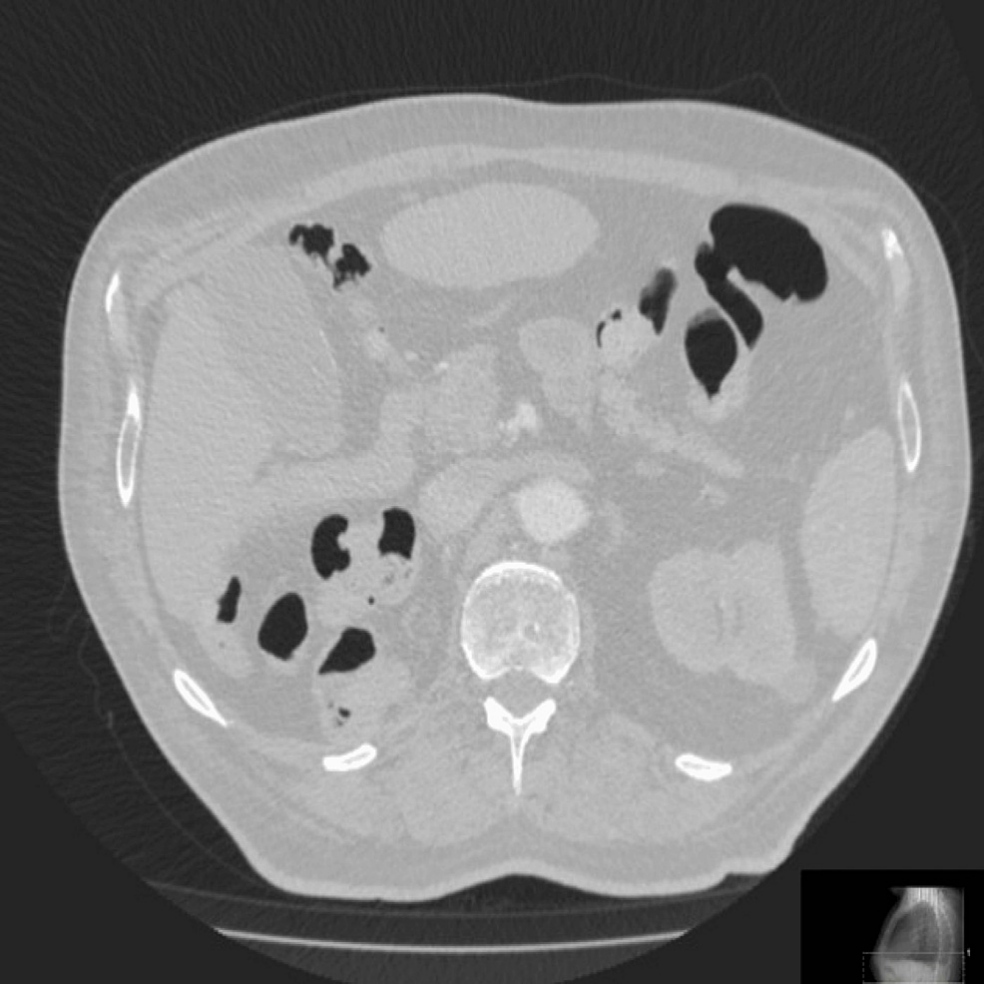
Case 1: Axial CT image in the lung window showing complete resolution of the extensive pneumoperitoneum.

At this time, a differential was made of pneumoperitoneum due to a leak at the gastroesophageal junction. However, following a discussion with the radiologist and a discussion with the tertiary centre, an impression of pneumoperitoneum from a recent right-sided nephrectomy was made. The patient was discharged home following conservative management and a repeat CT was requested which showed much resolution of massive pneumoperitoneum (Figure 3).

Case 2: pneumomediastinum

**Figure 4:**
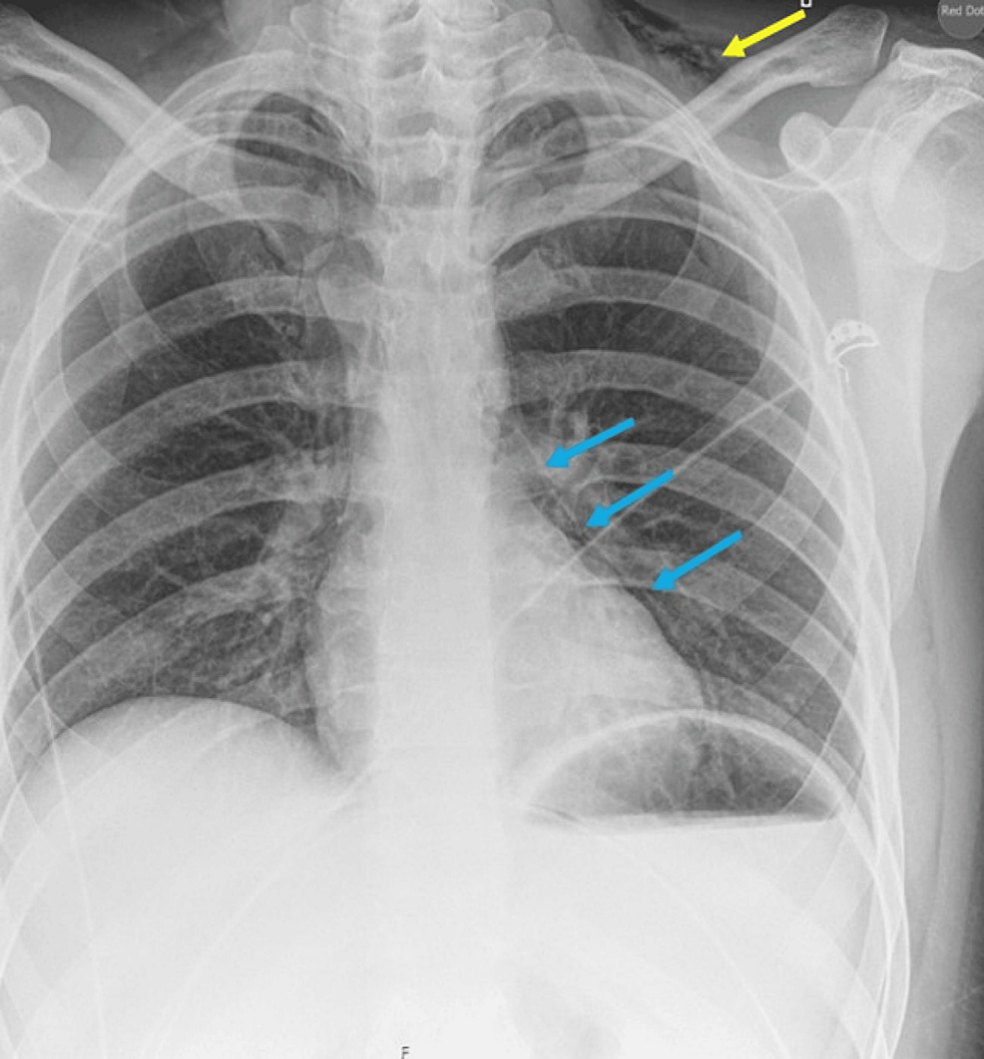
Case 2: Chest x-ray posteroanterior (PA) view with a query for pneumomediastinum (blue arrows) and possible left-sided subcutaneous emphysema along soft tissues of the neck (yellow arrow).

A 30-year-old non-smoker male with a BMI of 26 and a background history of type 1 DM with a history of five days of being generalised unwell with cough, nausea and vomiting presented to the ED. He noticed progressive worsening of his symptoms and had not been eating and drinking for two days and decided to omit subcutaneous insulin. His blood showed evidence of diabetic ketoacidosis (DKA), with the rise in inflammatory markers and he was started on intravenous fluids, antibiotics and sliding scale insulin. He additionally complained of vague discomfort in the left upper chest area. A chest X-ray was requested which suggested a query for left-sided pneumomediastinum along with left-sided small surgical emphysema along the soft tissues of the neck (Figure 4).

**Figure 5:**
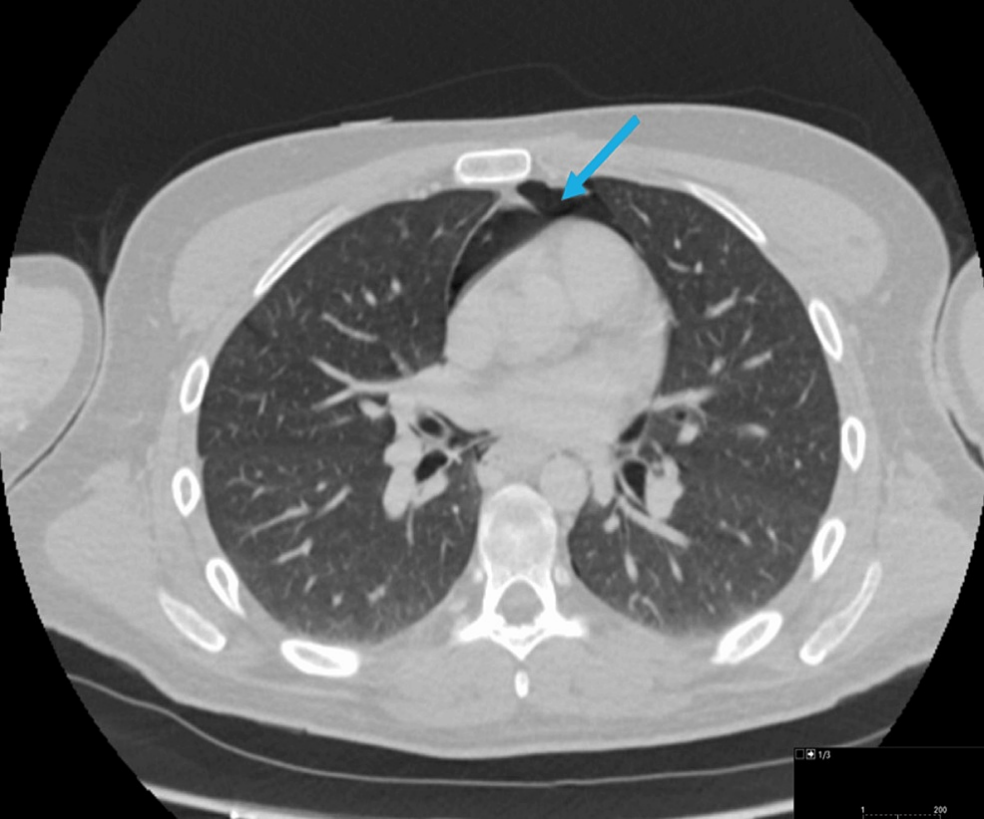
Case 2: Axial computed tomogram (CT) in lung window showing pneumomediastinum (blue arrow).

A CT scan was requested to characterise the extent of pneumomediastinum further and it showed pneumomediastinum (Figure 5).

**Figure 6:**
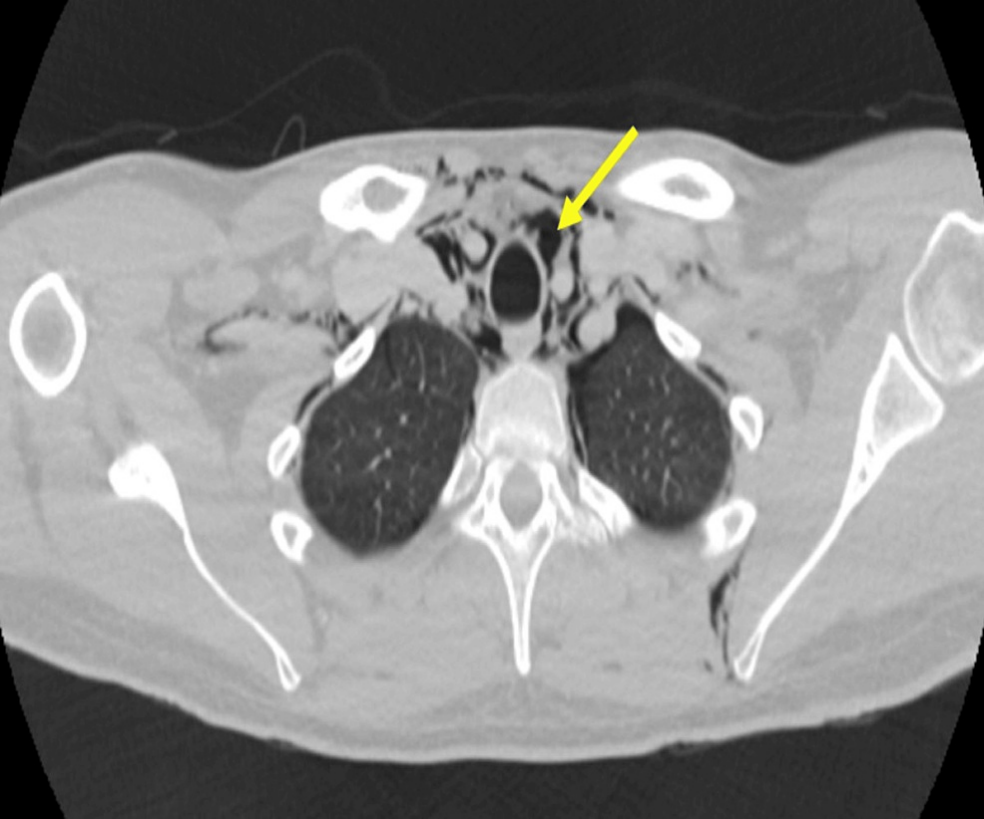
Case 2: Axial computed tomogram (CT) in the lung window showing a small left-sided pneumothorax (yellow arrow).

The CT scan also showed a small pneumothorax along the left side (Figure 6).

**Figure 7:**
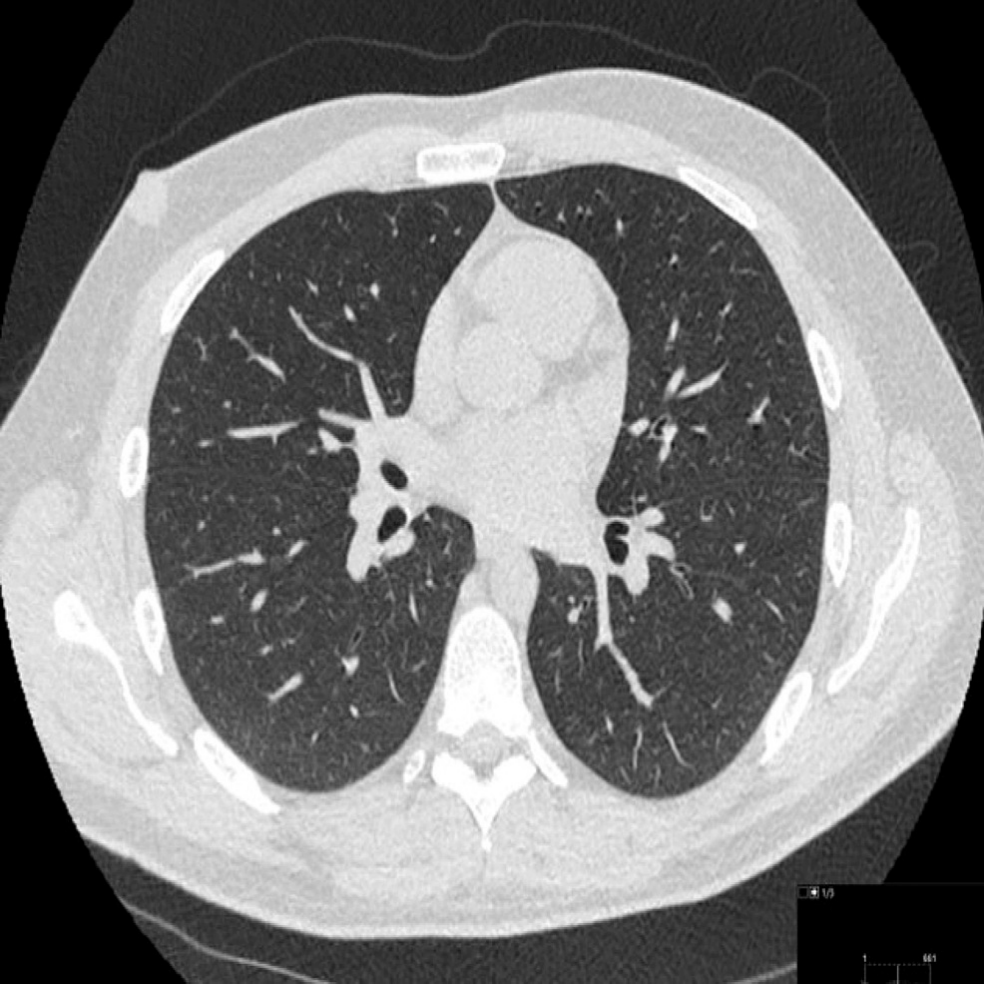
Case 2: Axial CT image in the lung window showing complete resolution of the extensive pneumomediastinum.

His case was discussed at the tertiary centre and advised conservative management. He responded well to DKA management and following review by the diabetic team discharged home in three days, with advice to seek medical advice if symptoms worsened and follow-up CT in three months which showed significant resolution (Figure 7).

## Discussion

The incidence of pneumomediastinum has been reported by Russo et al. as 1/44,500 attendances to the ED or 1/100,000 natural births. However, the incidence seems to be higher among children around 1/800 to 1/15,500 [8]. Jougon et al have reported the incidence to be around 1/25,000 in patients aged between 5 and 34 years [9]. Agut et al. have reported the majority of patients being male accounting for 76% of cases, as seen in our patients [6].

There are rare cases where air has been reported to dissect between the mediastinum and the spine causing cervicothoracolumbar pneumorrhachis defined as free air in the spinal canal [10].

Most cases of pneumomediastinum respond to conservative management and this is considered an acceptable practice although some patients may require management with low positive end-expiratory pressure ventilatory support [11]. Other supportive measures include analgesia, anti-anxiety medications, antitussives and supplemental oxygen support [12].

On the contrary, patients with pneumoperitoneum when managed with mechanical ventilation may develop abdominal compartment syndrome which may need to be treated with decompressive laparotomy [13]. The abdominal compartment syndrome has been defined as high intraabdominal pressure that causes end-organ compromise [14]. A fatality has been reported in a frail patient with massive retroperitoneal air post-intubation and flexible bronchoscopy, which led to the collapse of the inferior vena cava and left-sided intra-abdominal organs shift where the next of kin declined surgical decompression [15]. 

Lastly, most cases respond well to conservative supportive management. However, cases have been reported with symptoms two months post-incidence signifying periodic review with imaging if deemed necessary as we followed up with our patients to ensure resolution [16,17], but the long-term follow-up has not been reported perhaps due to relative scarcity of recurrence [6,18].

We report the case of a patient who was suspected to have pneumomediastinum following a chest X-ray on a background of having a cough. Following the iatrogenic presentation of pneumomediastinum post-robotic laparoscopic urological surgery. This emphasises the importance of history taking and examination and the use of imaging towards outlining the pathology assisting in outlining the management plan.

## Conclusions

Although our patients were managed via a conservative approach, it remains important to understand that fatalities have been reported in the literature. This highlights the need for prompt assessment to outline an appropriate management plan. We hope this case report will enable those involved in the management of patients at junior levels to be aware of noticing the observations or vital signs to assist in outlining the management plan in their day-to-day practice.

## References

[1] Lee CH (2010). Images in clinical medicine. Radiologic signs of pneumoperitoneum. N Engl J Med.

[2] Sureka B, Bansal K, Arora A (2015). Pneumoperitoneum: what to look for in a radiograph?. J Family Med Prim Care.

[3] Kadkhodaie H, Vaziri M (2008). Asymptomatic spontaneous pneumoperitoneum. Shiraz E-Med J.

[4] Kobashi Y, Okimoto N, Matsushima T, Soejima R (2002). Comparative study of mediastinal emphysema as determined by etiology. Intern Med.

[5] Sahni S, Verma S, Grullon J, Esquire A, Patel P, Talwar A (2013). Spontaneous pneumomediastinum: time for consensus. N Am J Med Sci.

[6] Agut A, Talavera J, Buendia A, Anson A, Santarelli G, Gomez S (2015). Imaging diagnosis-spontaneous pneumomediastinum secondary to primary pulmonary pathology in a dalmatian dog. Vet Radiol Ultrasound.

[7] Hamman L (1939). Spontaneous mediastinal emphysema. Bull Johns Hopkins Hosp.

[8] Russo A, Del Vecchio C, Zaottini A, Giangregorio C (2012). Role of emergency thoracic ultrasonography in spontaneous pneumomediastinum. two case report. G Chir.

[9] Jougon JB, Ballester M, Delcambre F, Mac Bride T, Dromer CE, Velly JF (2003). Assessment of spontaneous pneumomediastinum: experience with 12 patients. Ann Thorac Surg.

[10] Al-Mufarrej F, Gharagozloo F, Tempesta B, Margolis M (2009). Spontaneous cervicothoracolumbar pneumorrhachis, pneumomediastinum and pneumoperitoneum. Clin Respir J.

[11] Sathiyaseelan SL, Senthil N, Varadaraj P (2018). Case of pneumomediastinum due to alveolar rupture following endotracheal intubation. BMJ Case Rep.

[12] Kouritas VK, Papagiannopoulos K, Lazaridis G (2015). Pneumomediastinum. J Thorac Dis.

[13] Cadena M, Solano J, Caycedo N, Gomez D, Vinck EE, Quiroga P, Gaete P (2019). Tension pneumoperitoneum: case report of a rare form of acute abdominal compartment syndrome. Int J Surg Case Rep.

[14] Fagenholz P, Alam H Abdominal compartment syndrome. Critical Care Secrets.

[15] Po PL, Bai HF, Lin CH, Lin CC (2021). Pneumomediastinum and tension pneumoperitoneum following Bronchioloalveolar lavage in a mechanically ventilated patient. Respir Med Case Rep.

[16] Caceres M, Ali SZ, Braud R, Weiman D, Garrett HE Jr (2008). Spontaneous pneumomediastinum: a comparative study and review of the literature. Ann Thorac Surg.

[17] Gerazounis M, Athanassiadi K, Kalantzi N, Moustardas M (2003). Spontaneous pneumomediastinum: a rare benign entity. J Thorac Cardiovasc Surg.

[18] Koullias GJ, Korkolis DP, Wang XJ, Hammond GL (2004). Current assessment and management of spontaneous pneumomediastinum: experience in 24 adult patients. Eur J Cardiothorac Surg.

